# Maternal Obesity Doubles the Risk of Preeclampsia and Eclampsia: Post-COVID Changes in a Brazilian Cohort of 2.3 Million Hospitalizations

**DOI:** 10.3390/pathophysiology33030051

**Published:** 2026-07-15

**Authors:** Priscilla Vogt Fernandes de Souza, Maria Clara Salgado-Ramos, Jhenifer de Paiva Quadros, Juliana Cardoso Moraes, Daniel Luiz Santos da Silva, Alex Oliveira da Camara, Rafaella de Carvalho Cardoso, Hércules Rezende Freitas

**Affiliations:** 1 Health Informatics Laboratory (LabInfoS), Department of Integrated Medical Sciences, Faculty of Medical Sciences, State University of Rio de Janeiro, Rio de Janeiro 28905-320, RJ, Brazil; priscilla.vogt@animaeducacao.com.br (P.V.F.d.S.); mariaclarasalgadoramos@gmail.com (M.C.S.-R.); jheniferdpq@gmail.com (J.d.P.Q.); alex.nutrj@gmail.com (A.O.d.C.); 2 School of Health Sciences, IBMR University Center, Barra da Tijuca, Rio de Janeiro 22631-002, RJ, Brazil; julianacardosom1@gmail.com (J.C.M.); danielluis843@gmail.com (D.L.S.d.S.); rafaella.cardoso@ulife.com.br (R.d.C.C.); 3 Laboratory of Neuropharmacogenetics, Department of Pharmacology and Psychobiology, Roberto Alcântara Gomes Institute of Biology, State University of Rio de Janeiro, Vila Isabel, Rio de Janeiro 20551-030, RJ, Brazil

**Keywords:** eclampsia, preeclampsia, maternal obesity, hypertensive disorders of pregnancy, maternal health

## Abstract

**Background/Objectives:** Hypertensive disorders of pregnancy, particularly preeclampsia and eclampsia, are associated with high maternal and fetal morbidity and mortality, with maternal obesity representing a major, modifiable risk factor. This study aimed to estimate the association between obesity and preeclampsia/eclampsia (PEC) in obstetric hospitalizations in the state of São Paulo, Brazil, and to examine risk trends across maternal age groups and between two distinct temporal cohorts. **Methods:** This population-based observational study analyzed 2,338,934 obstetric hospitalizations using data from the Hospital Information System of the Unified Health System (SIH/SUS). PEC and obesity diagnoses were coded according to the International Classification of Diseases, 10th Revision (ICD-10). Logistic regression models were applied to estimate associations adjusted for maternal age, complemented by adjusted marginal predictions. **Results:** PEC occurred more than twice as often among women with obesity than without (5.4% vs. 2.2%; OR 2.13, 95% CI 1.84–2.47) and was associated with longer hospital stays (3.52 vs. 2.70 days) and higher costs (R$797.91 vs. R$602.69). The absolute risk difference between obese and non-obese women declined from 24.7% to 13.6% at age 20 between the 2017–2019 and 2023–2025 cohorts, while the relative risk gradient remained stable across all ages. **Conclusions:** Obesity is strongly associated with increased PEC risk, longer hospitalization, and higher costs. The observed attenuation in absolute risk differences in the most recent cohort reinforces the need for preventive interventions, nutritional surveillance, and public health policies aimed at reducing hypertensive complications during pregnancy for both obese and non-obese women.

## 1. Introduction

Hypertensive disorders of pregnancy (HDPs) encompass gestational hypertension, preeclampsia, eclampsia, chronic hypertension, and superimposed hypertensive forms. HDPs are recognized among the leading causes of maternal morbidity and mortality worldwide, especially in low- and middle-income countries [1]. From a global perspective, it is estimated that HDP affects between 5% and 10% of pregnancies, with variations related to geographic region, sociodemographic profile, and access to prenatal care. Eclampsia is the most severe manifestation of this spectrum, characterized by the occurrence of tonic–clonic seizures in women with preeclampsia (PE) and associated with a high risk of maternal and fetal complications. Among the modifiable risk factors of PE, maternal obesity has received increasing attention, particularly considering the continuous rise in prevalence among women of reproductive age [2,3]. A greater incidence of PE has been associated with the concomitant growth in overweight and obesity rates [3,4,5,6].

Contemporary evidence reinforces the association between maternal adiposity and HDP. In a recent meta-analysis, maternal adiposity indicators, including pre-pregnancy body mass index, subcutaneous fat thickness, waist circumference, and waist-to-hip ratio, were associated with higher odds of developing HDP, with odds ratios ranging from 1.67 to 3.08 [6]. Studies on adipose tissue distribution in the general population demonstrate that both subcutaneous adipose tissue (SAT) and visceral adipose tissue (VAT) are associated with metabolic risk factors [7]. Because both compartments are linked to metabolic and inflammatory changes, central obesity in early pregnancy may contribute directly to PE risk [8,9]. In large administrative databases, the clinical diagnosis of obesity is used as a practical indicator of this risk, and likely captures much of the risk attributable to central adiposity [10].

Recent Brazilian studies support the value of nutritional interventions and monitoring nutritional status during pregnancy. The ProcriAr Study (2023), conducted in the state of São Paulo, identified dietary patterns associated with excessive gestational weight gain (EGWG), demonstrating a higher probability of EGWG in women who were already overweight at the beginning of pregnancy, while the traditional Brazilian dietary pattern showed a protective effect [11]. The Brazilian Federation of Gynecology and Obstetrics Associations (FEBRASGO) guidelines propose standardized monitoring of gestational weight gain according to pre-pregnancy BMI, using specific curves and updated prenatal care tools [12]. Such recommendations are relevant to public health because they enable the early identification of pregnant women at higher risk, which supports earlier nutritional, behavioral, and hypertensive surveillance before clinical manifestation of conditions such as preeclampsia [13].

Even so, few Brazilian population-based studies have directly examined the association between pre-pregnancy obesity and PE, and evidence specific to eclampsia is harder still to find; most of the international literature addresses PE alone. What evidence exists comes mainly from clinical cohorts and meta-analyses conducted in North America, Europe, and East Asia [14,15]; data from public health systems in large, urbanized settings such as the state of São Paulo remain scarce. A separate, underexplored question is whether this association is stable over time. The COVID-19 pandemic disrupted obstetric care globally, affecting prenatal visits, lifestyle habits, comorbidity management, and health service organization [16,17], and SARS-CoV-2 infection during pregnancy was linked to increased severe maternal morbidity, including hypertensive complications and mortality, particularly early in the pandemic [14]. The biological link between obesity and PE is well established [8,10], but whether pandemic-era changes altered its magnitude, or shifted absolute risk levels at the population level, remains unknown. Few studies have examined the population epidemiology of hypertensive disorders of pregnancy in the post-pandemic period [18,19].

To fill these gaps, the present study uses population-based administrative data from the Hospital Information System of the Unified Health System (SIH/SUS) [20], which lets us evaluate the association between obesity and preeclampsia/eclampsia (PEC) under real-world conditions of public health service utilization and in one of the most populous and urbanized regions of Brazil. The comparison between two distinct temporal cohorts, corresponding to the pre-pandemic period (2017 to 2019) and the post-pandemic period (2023 to 2025), lets us test whether the magnitude of the association differs between the two periods [15,16]. Additionally, the estimation of adjusted marginal probabilities along the continuous maternal age range lets us examine how risk varies across maternal age [21,22].

Here, we analyzed the association between obesity and the occurrence of PEC in women of reproductive age in the state of São Paulo, using secondary data from obstetric hospitalizations from the SIH/SUS, comparing two distinct temporal cohorts flanking the COVID-19 pandemic and estimating adjusted marginal probabilities for maternal age. This design offers, to our knowledge, the first population-level quantification of how the absolute risk difference between obese and non-obese women has changed in Brazil.

## 2. Materials and Methods

### 2.1. Study Design

A population-based observational study was conducted using hospital admission records from the Hospital Information System of the Brazilian Unified Health System (SIH-SUS). Obstetric admissions of women between 10 and 55 years of age, residing in the state of São Paulo, were selected for two comparative temporal cohorts: 2017 to 2019 and 2023 to 2025.

### 2.2. Data Source

Microdata was obtained directly from the DataSUS database using the *microdatasus* package in R [20]. Files were downloaded, processed, and stored in parquet format to ensure efficient reading and data handling. All available clinical information was used, including primary and secondary diagnoses, demographic data, admission characteristics, costs, and hospital outcomes.

### 2.3. Data Processing

Diagnoses for each admission were coded according to the International Classification of Diseases, 10th revision (ICD-10). The primary diagnosis was standardized in uppercase letters and used as the reference for classifying admissions. Indicator variables were created for obstetric conditions (codes beginning with “O”), preeclampsia and eclampsia (O14 and O15), and obesity, identified by both the specific code E66 and code O99.2 (“Obesity complicating pregnancy, childbirth, or the puerperium”). An auxiliary function was developed to scan all diagnosis columns and increase sensitivity in detecting the presence of obesity.

Additional variables were derived for age in completed years, municipality of residence, race/ethnicity, length of hospital stay, and total admission cost. Severity outcomes included in-hospital death and number of days in the intensive care unit (ICU), extracted from specific database columns.

The primary dependent variable was the occurrence of PEC during the admission (binary). The exposure variable was the presence of obesity identified by ICD-10 code. Covariates included maternal age (in years, also categorized into age groups), municipality of residence, and year of admission.

### 2.4. Statistical Analysis

The association between obesity and PEC was estimated using logistic regression models with fixed effects for municipality of residence and year of admission, to control for spatial and temporal heterogeneity. Age was included as a continuous variable modeled with natural splines (4 degrees of freedom) to capture potential non-linear effects. Robust standard errors were adjusted for clustering at the municipality level.

Two pre-specified sensitivity analyses were conducted to assess the robustness of the primary estimates. First, hospitalizations with concurrent diagnoses of multiple gestation (ICD-10 O30, O31, O37) were excluded, given the distinct physiological risk profile associated with this condition. Second, recognizing that pre-existing hypertension and diabetes are recoded in Brazilian obstetric administrative data using pregnancy-specific ICD-10 categories (O10 for pre-existing hypertension complicating pregnancy; O24.0, O24.1, and O24.3 for pre-existing diabetes mellitus), direct covariate adjustment was not feasible due to complete separation between these codes and the PEC outcome within fixed-effect cells. As an alternative, admissions with any of these comorbidity codes were excluded from the analytic sample, restricting the analysis to women with no detected pre-existing vascular or metabolic risk. Both sensitivity analyses used the same model specification, fixed-effects structure, and marginal prediction approach as the primary analysis.

**Figure 1:**
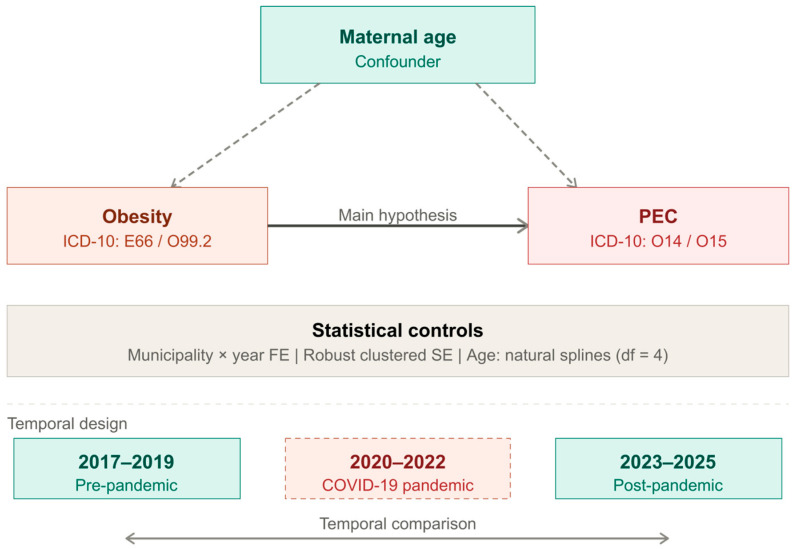
Conceptual framework and analytical model of the study. The upper panel presents a directed acyclic graph (DAG) depicting the causal structure of the analysis: maternal age acts as a confounder (dashed arrows) on both the exposure (obesity, ICD-10: E66/O99.2) and the outcome (preeclampsia/eclampsia, ICD-10: O14/O15), while the solid arrow represents the primary hypothesis under investigation. The statistical controls box summarizes the regression specification: fixed effects for municipality of residence and year of admission, robust standard errors clustered at the municipality level, and maternal age modeled as a continuous variable with natural splines (4 degrees of freedom). The lower panel illustrates the temporal design, showing the two analytical cohorts (2017–2019 and 2023–2025) flanking the excluded pandemic period (2020–2022). PEC: preeclampsia/eclampsia; FE: fixed effects; SE: standard errors.

Additionally, marginal predictions of the probability of the outcome were produced across different ages, stratified by obesity status. These predictions were accompanied by 95% confidence intervals obtained through parametric simulations based on the variance–covariance matrix of the fitted models (Figure 1). Predicted risk plots and summary tables were produced comparing women with and without obesity at reference ages of 20, 30, and 40 years. All analyses were performed in R (version 4.5.1, “Great Square Root”) within the RStudio integrated environment (version 2025.5.1.513, “Mariposa Orchid”). The R code was generated with the assistance of the Large Language Model Sonnet 4.6 (Claude Code, Anthropic, San Francisco, CA, USA) and reviewed completely by the authors. The complete commented R code can be found in Supplementary Code S1.

## 3. Results

### 3.1. Clinical, Obstetric, and Sociodemographic Characteristics

The clinical, obstetric, and sociodemographic characteristics of the women included in the study are described in Table 1. A total of 2,338,934 obstetric admissions were analyzed, of which 13,248 (0.6%) corresponded to women with obesity identified by ICD-10 code. The mean age of women with obesity was significantly higher compared to those without obesity (29.4 ± 6.5 vs. 26.9 ± 6.7 years; *p* < 0.001). The occurrence of PEC was observed at more than twice the rate among women with obesity (5.4%) compared to those without (2.2%; *p* < 0.001). Admissions of women with obesity also showed a longer mean length of hospital stay (3.5 ± 3.2 vs. 2.7 ± 2.3 days; *p* < 0.001) and higher costs (R$ 797.91 ± 521.29 vs. R$ 602.69 ± 367.23; *p* < 0.001). Regarding race/ethnicity, women with obesity more frequently self-identified as White (57.7% vs. 52.0%) or Black (8.3% vs. 6.8%), whereas a lower proportion self-identified as Brown (33.2% vs. 40.3%; *p* < 0.001).

### 3.2. Estimated Probabilities of PEC

**Figure 2:**
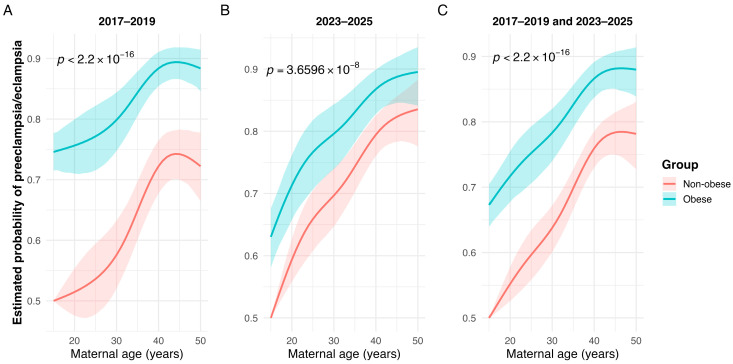
Estimated probability of PEC according to maternal age and obesity status across two temporal cohorts in the state of São Paulo. Values represent marginal predictions of PEC probability, obtained from logistic regression models with fixed effects for municipality of residence and year of admission and maternal age modeled as a continuous variable with natural splines (4 degrees of freedom), stratified by obesity (blue) and non-obesity (red). Ninety-five percent confidence intervals were derived through parametric simulation, drawing 1000 samples from the variance–covariance matrix of each fitted model. Panel (**A**) presents results for the 2017–2019 period, Panel (**B**) for the 2023–2025 period, and Panel (**C**) shows both cohorts overlaid for comparative purposes.

The values reported in Figure 2 are not raw observed proportions, but model-based marginal probabilities, derived from the logistic regression models described in Section 2.4. Across all maternal ages analyzed, obesity was associated with a higher predicted probability of PEC. Panel A (2017–2019) shows the largest separation between the two groups from age 30 onward, when the predicted probability among obese women exceeded 0.8 compared to values close to 0.6 among non-obese women. Panel B (2023–2025) shows the same gradient at a lower absolute magnitude, with obese women predicted at consistently higher probabilities across all ages. Panel C, overlaying both cohorts, shows the gap between obese and non-obese women persisting over time, with non-overlapping 95% confidence intervals at ages 30 and 40.

Table 2 presents the estimated mean probabilities of PEC in women with and without obesity according to maternal age and admission period. Across all ages and cohorts, obesity was associated with a higher risk of the outcome. Between 2017 and 2019, the absolute risk difference (ARD) ranged from 24.7% at age 20 to 16.3% at age 40, with adjusted odds ratios (ORs) remaining stable at approximately 2.96. In the 2023–2025 period, although the probabilities were lower in absolute terms, the association remained significant, with an ARD of 13.6% at age 20 and 6.6% at age 40, corresponding to an OR of approximately 1.77. When both cohorts were pooled, an intermediate ARD was observed (18.1% at age 20; 10.7% at age 40), with ORs close to 2.15 across all ages.

### 3.3. Sensitivity Analyses

Two sensitivity analyses confirmed the robustness of the primary findings. Exclusion of multiple-gestation admissions (SA1; n removed: approximately 5000 across the pooled cohort) produced estimates virtually identical to the main model, with ORs ranging from 2.95 at age 20 in the 2017–2019 cohort to 1.72 in the 2023–2025 cohort. Restriction to women with no coded pre-existing hypertension or diabetes (SA2) yielded slightly higher point estimates (OR 3.18 and 1.79 at age 20 in the pre- and post-pandemic cohorts, respectively), consistent with the partial capture of obesity-related vascular risk through alternative ICD-10 coding pathways in women with pre-existing comorbidities. The direction, magnitude, and temporal attenuation pattern of the obesity–PEC association were unchanged across both sensitivity analyses (Supplementary Table S1).

## 4. Discussion

The results of the present study demonstrated a strong and consistent association between maternal obesity and PEC, with obese pregnant women presenting substantially higher absolute probabilities and relative risks of PEC in both evaluated periods. The odds of the outcome were two to three times higher among women with obesity, regardless of maternal age. This corroborates Khan and colleagues (2023), who identified obesity as one of the most consistent risk factors for hypertensive complications in pregnancy [21], and supports a role for metabolic dysfunction in PEC pathophysiology. The increase in mean length of hospital stays and hospitalization costs observed in the group of women with obesity further reflects the clinical and economic burden of this condition on the health system.

A noteworthy aspect was the comparison between the pre-pandemic (2017–2019) and post-pandemic (2023–2025) periods, in which lower absolute PEC probabilities were observed in the more recent cohort despite the persistence of the association with obesity. During the COVID-19 pandemic, studies conducted in Brazil and other countries reported high prevalences of preeclampsia, particularly among infected pregnant women, reaching rates of up to 11% in hospital cohorts [14,16]; obesity was one of the factors most strongly associated with worsening the condition, along with viral infection and chronic hypertension [8,15]. In the subsequent period, mass immunization and heightened obstetric surveillance appear to have contributed to a decline in absolute risks [19].

Two explanations deserve particular attention, since both act directly on which admissions enter the SIH-SUS records in the first place. First, admission thresholds for obstetric hospitalization may have shifted between periods: if lower-acuity admissions became relatively less common in 2023–2025, the hospitalized population in that cohort would be enriched for more severe presentations in both exposure groups, compressing the absolute gap between obese and non-obese women without any change in the underlying biological risk. Second, an expansion of prenatal surveillance intensity, including more frequent blood pressure and proteinuria screening and broader telemedicine follow-up, could allow earlier detection and outpatient management of milder hypertensive disorders, keeping some cases that would previously have required hospitalization out of the admission records entirely. Beyond these two mechanisms, other non-mutually exclusive factors remain plausible, including changes in prenatal care organization, shifts in the demographic and behavioral profile of pregnant women, such as maternal age patterns, nutritional status, and cardiometabolic health [17], rising obesity prevalence among women of reproductive age [10], variations in obesity coding practices in administrative databases, and changes in exposure to SARS-CoV-2 infection and vaccination coverage over time [16,19].

The relative risk gradient between obese and non-obese women persisted across both cohorts even as absolute risks declined, consistent with data reported by Matsuo and colleagues (2023) [14]. Future longitudinal studies using individual-level clinical data will be needed to clarify which of these factors most plausibly contributed to the temporal pattern identified here.

**Figure 3:**
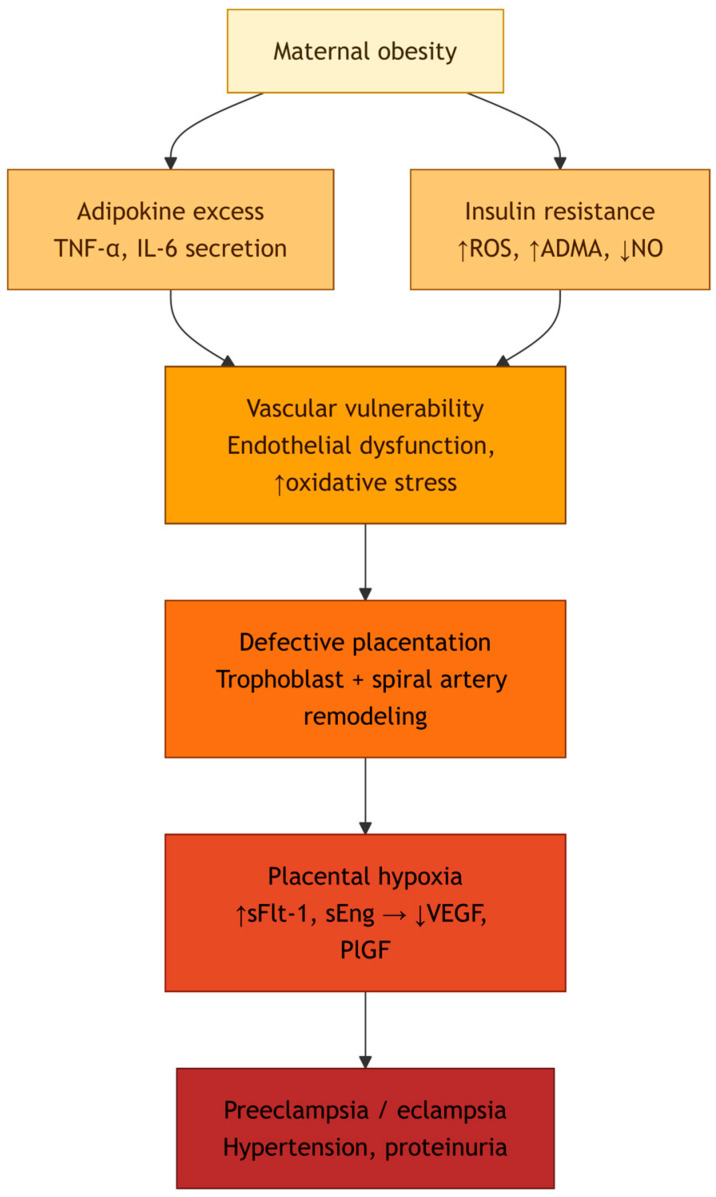
Proposed mechanistic cascade linking maternal obesity to preeclampsia/eclampsia (PEC). The two upstream pathways, adipokine excess and insulin resistance, converge on vascular vulnerability, triggering defective placentation, placental hypoxia, and antiangiogenic factor release, culminating in the clinical manifestations of PEC. This cascade is a proposed, literature-derived schematic intended to summarize plausible biological pathways; it has not been tested or validated with the administrative data used in this study. ADMA: asymmetric dimethylarginine; NO: nitric oxide; ROS: reactive oxygen species; sFlt-1: soluble fms-like tyrosine kinase-1; sEng: soluble endoglin; VEGF: vascular endothelial growth factor; PlGF: placental growth factor. Based on Lopez-Jaramillo et al. [9] and Roberts et al. [23].

From a pathophysiological perspective, the association between obesity and PEC observed here is consistent with a well-described biological pathway, although this pathway cannot be tested directly with SIH-SUS administrative data. Maternal obesity generates a persistent pro-inflammatory and insulin-resistant state, marked by excess secretion of adipokines and inflammatory cytokines (TNF-α, IL-6, leptin) alongside reduced adiponectin, together with increased reactive oxygen species and elevated ADMA, an endogenous inhibitor of nitric oxide synthesis [7,9]. This combination promotes endothelial dysfunction and vascular vulnerability, which has been linked to defective trophoblast invasion, inadequate spiral artery remodeling, and placental hypoxia. Placental hypoxia is thought to intensify the release of antiangiogenic mediators such as sFlt-1 and sEng, which antagonize VEGF and PlGF and are considered central to the clinical manifestations of PEC [23]. Reduced adiponectin signaling has also been linked to lower kisspeptin expression, a proposed additional mechanism compromising placental vascular remodeling [24]. Figure 3 summarizes this proposed cascade, from maternal obesity to the clinical manifestations of PEC.

The racial and ethnic differences observed in this cohort, with higher proportions of White and Black women among those with obesity and a lower proportion of Brown women, are consistent with broader evidence that racial and socioeconomic inequalities shape access to prenatal care and the management of hypertensive conditions in pregnancy [22,25]. Because obesity remained a strong risk factor for PEC in both cohorts, preventive strategies targeting women of reproductive age remain a priority. Weight management, preconception care, regular physical activity, and early identification of cardiometabolic risk factors may all help reduce adverse maternal outcomes. Given the rising global prevalence of obesity among women of reproductive age, further research into the mechanisms driving PEC in non-obese women is also needed, since obesity alone does not account for all cases. More broadly, our findings support screening for obesity-related risk in primary care as a practical measure to reduce obstetric complications and narrow inequalities in maternal health.

Several limitations inherent to the use of secondary administrative data warrant consideration. Obesity identification relies on ICD-10 coding in hospital records, which is subject to systematic undercoding in Brazilian obstetric settings: women with obesity who receive no corresponding diagnosis code are classified as non-exposed, introducing non-differential misclassification that biases the estimated odds ratios toward the null. The reported OR of 2.13 therefore likely underestimates the true magnitude of the association.

Continuous body mass index, gestational weight gain, and adipose tissue compartmentalization (including visceral and subcutaneous depots most directly implicated in PEC pathophysiology) cannot be recovered from SIH-SUS records, and women with excess central adiposity below formal diagnostic thresholds are similarly misclassified as non-exposed, contributing further to conservative effect estimates. Pre-existing chronic hypertension and diabetes mellitus are recoded in Brazilian obstetric administrative data as O10 and O24.0–O24.3 respectively, categories that are operationally near-mutually exclusive with the PEC outcome codes within the fixed-effect structure; direct covariate adjustment was therefore not feasible, and while the restriction-based sensitivity analysis addressed this analytically, residual confounding from these conditions in the main model cannot be fully excluded.

Information on antihypertensive, antidiabetic, and immunomodulatory drug therapy is not recorded in SIH-SUS and cannot be approximated from available fields. SARS-CoV-2 infection history is likewise unavailable for all women in the dataset; although the study design excluded the active pandemic period from both cohorts, prior infection among women admitted during 2023–2025 may have modified individual risk profiles in ways these data cannot address. Despite these constraints, the population-level scale of the dataset, the use of fixed-effects models with municipality-level clustering, and the consistency of primary estimates across two pre-specified sensitivity analyses collectively support the robustness of the main findings.

## 5. Conclusions

The most pertinent feature of these results is not the obesity–PEC association itself, which is well established, but its temporal behavior across cohorts. The absolute risk difference at age 20 declined from 24.7% in 2017–2019 to 13.6% in 2023–2025, with odds ratios falling in parallel from 2.96 to 1.77, a pattern consistent across all ages and confirmed by both sensitivity analyses. The mechanisms underlying this attenuation cannot be determined from administrative data alone; changes in prenatal care organization, hospitalization thresholds, population health behaviors, and coding practices over time are each plausible contributors, but distinguishing among them will require prospective studies with individual-level clinical data. What the data establish unambiguously is that even after this attenuation, obesity retained a strong and independent association with PEC in the most recent cohort, and an ARD of 13.6% at age 20 represents a clinically consequential disparity. Sustained investment in preconception weight management, early nutritional surveillance, and obesity-aware prenatal pathways therefore remains a structural priority for reducing hypertensive complications of pregnancy in Brazil, irrespective of how the temporal dynamics continue to evolve.

## Figures and Tables

**Table 1:** Clinical, obstetric, and sociodemographic characteristics of pregnant women according to nutritional status (2017–2019 and 2023–2025).

Variable	Overall	Non-Obese	Obese	*p*-Value
Absolute frequency	2,338,934	2,325,686	13,248	-
Age (mean ± SD)	26.86 (6.72)	26.85 (6.72)	29.42 (6.52)	<0.001 *
PEC (%)	51,323 (2.2)	50,609 (2.2)	714 (5.4)	<0.001 ^†^
Total cost (mean ± SD)	603.79 (368.58)	602.69 (367.23)	797.91 (521.29)	<0.001 *
Length of stay (mean ± SD)	2.71 (2.29)	2.70 (2.29)	3.52 (3.23)	<0.001 *
Race/ethnicity (%)				<0.001 ^Δ^
White	1,122,311 (52.1)	1,114,759 (52.0)	7552 (57.7)	-
Black	145,998 (6.8)	144,909 (6.8)	1089 (8.3)	-
Brown	867,254 (40.2)	862,914 (40.3)	4340 (33.2)	-
Asian	19,350 (0.9)	19,249 (0.9)	101 (0.8)	-
Indigenous	875 (0.0)	865 (0.0)	10 (0.1)	-

Notes: PEC—Preeclampsia/Eclampsia. * Student’s *t*-test; ^†^ Fisher’s exact test; ^Δ^ Chi-square test. Length of stay measured in days.

**Table 2:** Estimated mean probability of PEC in women with and without obesity according to maternal age and admission period.

Period	Age	Non-Obese (Mean [CI 95%])	Obese (Mean [CI 95%])	ARD (%)	OR
2017–2019	20	0.50 [0.50–0.50]	0.75 [0.71–0.78]	24.65	2962
	30	0.61 [0.56–0.66]	0.82 [0.77–0.86]	21.18	2966
	40	0.72 [0.66–0.77]	0.88 [0.84–0.91]	16.28	2964
2023–2025	20	0.50 [0.50–0.50]	0.64 [0.59–0.68]	13.63	1762
	30	0.70 [0.65–0.75]	0.80 [0.74–0.85]	10.26	1767
	40	0.82 [0.74–0.88]	0.89 [0.82–0.94]	6.57	1778
2017–2025	20	0.50 [0.50–0.50]	0.68 [0.65–0.71]	18.08	2141
	30	0.65 [0.62–0.68]	0.80 [0.76–0.83]	14.78	2145
	40	0.77 [0.71–0.82]	0.88 [0.83–0.91]	10.71	2147

Notes: ARD—Absolute risk difference; OR—Odds ratio.

## Data Availability

The data presented in this study are available in [DATASUS] at [https://datasus.saude.gov.br/informacoes-de-saude-tabnet/], reference number [Microdatasus]. These data were derived from the following resources available in the public domain: [https://doi.org/10.1590/0102-311X00032419].

## Supplementary Materials

The following supporting information can be downloaded at https://www.mdpi.com/article/10.3390/pathophysiology33030051/s1, Code S1: Commented R code for reproducibility; Table S1: Sensitivity analyses: estimated probability of preeclampsia/eclampsia (PEC) by age, obesity status, cohort, and analytic restriction.

## Abbreviations

The following abbreviations are used in this manuscript:

ADMA	Asymmetric dimethylarginine
ARD	Absolute risk difference
BMI	Body mass index
CI	Confidence interval
COVID-19	Coronavirus disease 2019
DataSUS	Brazilian Health Informatics Department
EGWG	Excessive gestational weight gain
HDP	Hypertensive disorders of pregnancy
ICD-10	International Classification of Diseases, 10th revision
ICU	Intensive care unit
IL-6	Interleukin-6
NO	Nitric oxide
OR	Odds ratio
PE	Preeclampsia
PEC	Preeclampsia/eclampsia
PlGF	Placental growth factor
ROS	Reactive oxygen species
SARS-CoV-2	Severe acute respiratory syndrome coronavirus 2
SD	Standard deviation
sFlt-1	Soluble fms-like tyrosine kinase-1
sEng	Soluble endoglin
SIH-SUS	Hospital Information System of the Brazilian Unified Health System
TNF-α	Tumor necrosis factor alpha
VEGF	Vascular endothelial growth factor

## References

[1] Bilano V.L., Ota E., Ganchimeg T., Mori R., Souza J.P. (2014). Risk Factors of Pre-Eclampsia/Eclampsia and Its Adverse Outcomes in Low- and Middle-Income Countries: A WHO Secondary Analysis. PLoS ONE.

[2] Horgan R., Hage Diab Y., Costantine M., Saade G., Sibai B. (2025). Diagnosis of Hypertensive Disorders in Pregnancy. Am. J. Obstet. Gynecol. MFM.

[3] Kent L., McGirr M., Eastwood K.-A. (2024). Global Trends in Prevalence of Maternal Overweight and Obesity: A Systematic Review and Meta-Analysis of Routinely Collected Data Retrospective Cohorts. Int. J. Popul. Data Sci..

[4] Lourenço J., Guedes-Martins L. (2025). Pathophysiology of Maternal Obesity and Hypertension in Pregnancy. J. Cardiovasc. Dev. Dis..

[5] Nikolova Z., Sandeva M., Uchikova E., Kirkova-Bogdanova A., Taneva D., Vladimirova M., Georgieva L. (2025). Impact of Maternal Overweight and Obesity on Pregnancy Outcomes Following Cesarean Delivery: A Retrospective Cohort Study. Healthcare.

[6] Xiang C., Sui L., Ding X., Cao M., Li G., Du Z. (2024). Maternal Adiposity Measures and Hypertensive Disorders of Pregnancy: A Meta-Analysis. BMC Pregnancy Childbirth.

[7] Abraham T., Romani A.M.P. (2022). The Relationship between Obesity and Pre-Eclampsia: Incidental Risks and Identification of Potential Biomarkers for Pre-Eclampsia. Cells.

[8] Zhang Y., Lu M., Yi Y., Xia L., Zhang R., Li C., Liu P. (2024). Influence of Maternal Body Mass Index on Pregnancy Complications and Outcomes: A Systematic Review and Meta-Analysis. Front. Endocrinol..

[9] Lopez-Jaramillo P., Barajas J., Rueda-Quijano S.M., Lopez-Lopez C., Felix C. (2018). Obesity and Preeclampsia: Common Pathophysiological Mechanisms. Front. Physiol..

[10] Poston L., Caleyachetty R., Cnattingius S., Corvalán C., Uauy R., Herring S., Gillman M.W. (2016). Preconceptional and Maternal Obesity: Epidemiology and Health Consequences. Lancet Diabetes Endocrinol..

[11] Saldiva S.R.D.M., De Arruda Neta A.d.C.P., Teixeira J.A., Peres S.V., Marchioni D.M.L., Carvalho M.A., Vieira S.E., Francisco R.P.V. (2022). Dietary Pattern Influences Gestational Weight Gain: Results from the ProcriAr Cohort Study-São Paulo, Brazil. Nutrients.

[12] Surita F.G.d.C., Souza R.T., Carrilho T.R.B., Hsu L.d.P.R., Mattar R., Kac G. (2023). Guidelines on How to Monitor Gestational Weight Gain during Antenatal Care. Rev. Bras. Ginecol. Obstet..

[13] Geus L.M.M.d., Maciel C.S., Burda I.C.A., Daros S.J., Batistel S., Martins T.C.A., Ferreira V.A., Ditterich R.G. (2011). A importância na inserção do nutricionista na Estratégia Saúde da Família. Ciênc. Saúde Coletiva.

[14] Matsuo K., Green J.M., Herrman S.A., Mandelbaum R.S., Ouzounian J.G. (2023). Severe Maternal Morbidity and Mortality of Pregnant Patients With COVID-19 Infection During the Early Pandemic Period in the US. JAMA Netw. Open.

[15] Xiong Y., Chen J., Wu Y., Zhao P., Liao M., Guo J., Liu C., Zheng M., Ren Y., Zou K. (2025). The Effect of Maternal Pre-Pregnancy Body Mass Index on Hypertensive Disorders of Pregnancy (HDP): A Systematic Review and Dose-Response Meta-Analysis of Cohort Studies Involving 50 Million Pregnancies. EClinicalMedicine.

[16] Molina R.L., Tsai T.C., Dai D., Soto M., Rosenthal N., Orav E.J., Figueroa J.F. (2022). Comparison of Pregnancy and Birth Outcomes Before vs During the COVID-19 Pandemic. JAMA Netw. Open.

[17] Ferrara A., Greenberg M., Zhu Y., Avalos L.A., Ngo A., Shan J., Hedderson M.M., Quesenberry C.P. (2023). Prenatal Health Care Outcomes Before and During the COVID-19 Pandemic Among Pregnant Individuals and Their Newborns in an Integrated US Health System. JAMA Netw. Open.

[18] Sobieray N.L., Carvalho N.S., Klas C.F., Furuie I.N., Chiste J.A., Fugaça C.A., Longo J.S., Oliveira J.D., Padilha S.L. (2024). Preeclampsia in Pregnant Women with COVID-19: A Prospective Cohort Study from Two Tertiary Hospitals in Southern Brazil. PeerJ.

[19] Iannaccone A., Gellhaus A., Reisch B., Dzietko M., Schmidt B., Mavarani L., Kraft K., Andresen K., Kimmig R., Pecks U. (2024). The Importance of Vaccination, Variants and Time Point of SARS-CoV-2 Infection in Pregnancy for Stillbirth and Preterm Birth Risk: An Analysis of the CRONOS Register Study. J. Clin. Med..

[20] Saldanha R.d.F., Bastos R.R., Barcellos C. (2019). Microdatasus: Pacote para download e pré-processamento de microdados do Departamento de Informática do SUS (DATASUS). Cad. Saúde Pública.

[21] Khan S.S., Petito L.C., Huang X., Harrington K., McNeil R.B., Bello N.A., Bairey Merz C.N., Miller E.C., Ravi R., Scifres C. (2023). Body Mass Index, Adverse Pregnancy Outcomes, and Cardiovascular Disease Risk. Circ. Res..

[22] Xie J., Yan Y., Ye Z., Wu Y., Yu Y., Sun Y., Rong S., Santillan D.A., Ryckman K., Snetselaar L.G. (2025). Racial/Ethnic Disparities in the Association of Maternal Diabetes and Obesity with Risk of Preterm Birth among 17 Million Mother-Infant Pairs in the United States: A Population-Based Cohort Study. BMC Pregnancy Childbirth.

[23] Roberts J.M., Bodnar L.M., Patrick T.E., Powers R.W. (2011). The Role of Obesity in Preeclampsia. Pregnancy Hypertens. Int. J. Women’s Cardiovasc. Health.

[24] Carrilho I., Mariana M., Cairrao E. (2025). Adiponectin as a Biomarker of Preeclampsia: A Systematic Review. Reprod. Med..

[25] Kracht C.L., Harville E.W., Cohen N.L., Sutton E.F., Kebbe M., Redman L.M. (2025). Racial Disparities in Gestational Weight Gain and Adverse Pregnancy Outcomes among Black and White Pregnant People with Obesity. Obesity.

