# Supplementary material for: Maternal Obesity Doubles the Risk of Preeclampsia and Eclampsia: Post-COVID Changes in a Brazilian Cohort of 2.3 Million Hospitalizations
Source: Pathophysiology. 2026 Jul 15;33(3):51. doi: 10.3390/pathophysiology33030051 (PMC13398326; doi:10.3390/pathophysiology33030051)
Supplement: Supplementary file 1 [file pathophysiology-33-00051-s001.zip › Table S1_sensitivity_analyses_final.pdf]

Supplementary Table S1. Sensitivity analyses: estimated probability of preeclampsia/eclampsia (PEC) by age, obesity status, cohort, and analytic restriction.

Analysis	Period	Age (years)	Non-obese (mean [95% CI])	Obese (mean [95% CI])	ARD (%)	OR
SA1: no multiple gestation	2017–2019	20	0.500 [0.500–0.500]	0.746 [0.712–0.777]	24.60	2.953
		30	0.609 [0.562–0.657]	0.820 [0.776–0.860]	21.09	2.957
		40	0.719 [0.659–0.775]	0.882 [0.845–0.915]	16.31	2.953
	2023–2025	20	0.500 [0.500–0.500]	0.631 [0.587–0.678]	13.08	1.720
		30	0.718 [0.673–0.758]	0.813 [0.762–0.857]	9.43	1.724
		40	0.835 [0.774–0.885]	0.896 [0.840–0.935]	6.02	1.732
	2017–2025	20	0.500 [0.500–0.500]	0.673 [0.638–0.705]	17.28	2.065
		30	0.665 [0.632–0.697]	0.803 [0.766–0.840]	13.78	2.068
		40	0.779 [0.725–0.826]	0.878 [0.838–0.912]	9.92	2.070
SA2: no hypertension/diabetes	2017–2019	20	0.500 [0.500–0.500]	0.759 [0.726–0.791]	25.94	3.175
		30	0.608 [0.559–0.658]	0.830 [0.786–0.869]	22.16	3.181
		40	0.728 [0.669–0.784]	0.893 [0.859–0.923]	16.59	3.176
	2023–2025	20	0.500 [0.500–0.500]	0.640 [0.589–0.689]	13.99	1.792
		30	0.723 [0.676–0.764]	0.822 [0.769–0.867]	9.89	1.797
		40	0.847 [0.786–0.892]	0.907 [0.853–0.945]	5.95	1.808
	2017–2025	20	0.500 [0.500–0.500]	0.684 [0.648–0.719]	18.42	2.177
		30	0.666 [0.630–0.699]	0.812 [0.772–0.846]	14.55	2.179
		40	0.790 [0.744–0.834]	0.890 [0.853–0.920]	10.02	2.181

SA1 excludes hospitalizations with concurrent multiple-gestation diagnoses (ICD-10 O30, O31, O37). SA2 excludes women with coded pre-existing hypertension (O10) or diabetes mellitus (O24.0, O24.1, O24.3). Values represent marginal predictions derived from logistic regression models with fixed effects for municipality of residence and year of admission, with 95% confidence intervals obtained via parametric simulation (n = 1,000 draws). ARD: absolute risk difference; OR: odds ratio.
